# Primary and secondary angiosarcomas: a comparative single-center analysis

**DOI:** 10.1186/s13569-015-0028-9

**Published:** 2015-05-23

**Authors:** Thorsten Hillenbrand, Franka Menge, Peter Hohenberger, Bernd Kasper

**Affiliations:** Sarcoma Unit, Interdisciplinary Tumor Center Mannheim, Mannheim University Medical Center, University of Heidelberg, Theodor-Kutzer-Ufer 1-3, 68167 Mannheim, Germany

**Keywords:** Primary angiosarcoma, Secondary angiosarcoma, Chemotherapy, Targeted therapy, Outcome

## Abstract

**Background:**

Angiosarcomas (AS) are rare vascular malignancies. They are subdivided into primary (PAS) and secondary angiosarcomas (SAS). The objective was to compare the characteristics of AS subtypes.

**Methods:**

Eighteen PAS and ten SAS patients treated at our institution between 2004 and 2012 were included in this study.

**Results:**

Median age of PAS and SAS patients was 52.9 and 64.2 years, respectively (p = 0.1448). The percentage of women was 27.8% for PAS, but 80.0% for SAS (p = 0.0163). While PAS occurred throughout the body, the majority of SAS arose from the breast (p = 0.0012). All SAS were radiation-induced with a median latency of 7.7 years. The majority of patients with PAS and SAS underwent surgery as primary or recurrence treatment (p > 0.95). Local recurrence was developed by 27.8% of PAS and 50.0% of SAS (p = 0.4119). 61.1% of PAS metastasized, but only 40.0% of SAS (p = 0.4328). Median overall survival for PAS and SAS was 19 and 57 months, respectively (p = 0.2306).

**Conclusion:**

Radical surgery remains the mainstay of both primary and recurrence treatment. SAS show a high local recurrence rate, while PAS tend towards developing early metastases. Overall, prognosis is poor for both groups.

## Background

Angiosarcomas (AS) are rare and aggressive malignancies representing about 2% of all adult soft tissue sarcomas [1]. They arise from endothelial cells of blood vessels or lymphatics either sporadically as primary neoplasms or secondary to chronic lymphedema or previous irradiation [2]. The latter constitutes an increasing complication following breast conserving surgery and radiotherapy in patients with breast cancer [3, 4]. Over 200 cases of radiation-induced AS of the breast are currently known in literature [5]. AS can occur throughout the body: most commonly in the head and neck area, followed by breasts and extremities. The remainder arise from different localizations like the liver, the heart and the bone [6]. The two conditions are similar in terms of histopathological features and immunohistochemical markers [7]. Secondary AS (SAS) differentiate from primary AS (PAS) in their pathogenesis by showing high level amplifications of MYC as well as FLT4 (VEGFR3) [8, 9]. Evidence-based recommendations are missing for the treatment of AS. Radical surgical en bloc resection with negative margins (R0) is the primary therapy for a potentially curable localized disease [10–12]. When indicated, surgery should be completed by adjuvant radiotherapy to prevent local recurrence [13, 14]. Inoperable, locally advanced or metastatic AS are treated by cytotoxic chemotherapy. Some clinical trials displayed that doxorubicin-based regimens and paclitaxel are two of the most active agents [15–17]. Furthermore, molecularly targeted therapy, in particular antiangiogenic therapy, constitutes a new option of treatment. Sorafenib was identified as an active agent against AS for instance [18]. Despite all therapeutic efforts, the patients’ prognosis is still unfavorable [19, 20]. There is a relatively small amount of knowledge about the similarities and differences between the two subtypes of AS. As the objective of this retrospective study, the patient and tumor characteristics, treatment and outcome of the two different types of AS were analyzed.

## Methods

### Patients

All adult patients with a confirmed pathohistological diagnosis of AS were identified from the Sarcoma Unit of the Interdisciplinary Tumor Center of the University Medical Center in Mannheim between 2004 and 2012. The study population consisted of 28 patients [13 women (46.4%), 15 men (53.6%)]. Acquisition of clinical data was obtained from the medical records. Patient and tumor characteristics including gender, age at diagnosis, subtype, tumor site, tumor-related symptoms, as well as metastasis at initial diagnosis, treatment, pattern of recurrence, occurrence of metastasis, date of last follow-up and survival were recorded and analyzed. In case of SAS, both type and age at diagnosis of pre-existing condition, latent time interval from radiotherapy to diagnosis of SAS and dosage of radiation were reviewed. This retrospective study was approved by the local ethics committee.

### Statistical analysis

Progression-free survival (PFS) was defined as the time interval from the pathohistological diagnosis of AS until the time of first progression (local recurrence or metastasis) or the sarcoma-related death. Patients were censored at the last time of follow-up if they did not experience any disease progression or death. Overall survival (OS) was defined as the period of time from the pathohistological diagnosis until the patient’s death. Patients were censored if they were still alive at the last follow-up. PFS and OS were calculated by using the method of Kaplan and Meier. Comparison of survival curves were performed by log-rank tests. Differences between the two AS subtypes were evaluated by t tests or Fisher’s exact tests. StatXact 9.0 (Cytel Studios 2012, Cambridge, MA, USA) and SAS 9.2 (SAS Institute Inc. 2013, Cary, NC, USA) were used for the statistical analyses. Statistical level of significance was set at α = 0.05.

## Results

### Patients and clinical presentation

Patient and tumor characteristics of PAS and SAS are shown in Table 1. From 2004 to 2012, a total of 28 patients with AS were identified. Of these, 18 patients were diagnosed with PAS (64.3%) and ten patients with SAS (35.7%). The majority of patients with PAS were represented by males (n = 13; 72.2%) while patients with SAS were dominated by females (n = 8; 80%). Fisher’s exact test revealed statistical significance in gender distribution between the two groups (p = 0.0163). While PAS developed de novo, all patients with SAS had a previous history of radiotherapy with a median dose of 56 Gy (range 50.0–60.4 Gy). The most frequent condition for which patients were irradiated was breast cancer (n = 8; 80%). Further conditions were chronic myeloid leukemia (n = 1; 10%) and thyroid gland autonomy (n = 1; 10%). The median age at the time of pre-existing diagnosis was 56.8 years (range 18.5–71.9 years). The median latent time interval from radiotherapy to diagnosis of SAS was 7.7 years (range 4.4–33.5 years). The majority of PAS occurred in deep soft tissue or internal organs (n = 12; 66.7%). Overall, the most common primary tumor sites were bone (n = 4; 22.2%), skin (n = 4; 22.2%), heart (n = 3; 16.7%) and breast (n = 2; 11.1%). In contrast, only 20% of SAS developed in internal organs [thyroid gland (n = 1), liver (n = 1)] and 80% (n = 8) in the breast or chest wall. The distribution of tumor localization showed a statistically significant difference (p = 0.0012).

**Table 1:** Patient, tumor, therapy and recurrence characteristics of PAS and SAS

Characteristic	Primary angiosarcomas		Secondary angiosarcomas		p value
	n	%	n	%	
Total number of patients	18	64.3	10	35.7	
Male	13	72.2	2	20	0.0163
Female	5	27.8	8	80	
Age at initial diagnosis, median	52.9 years		64.2 years		0.1448
Primary tumor site					0.0012
Superficial soft tissue or skin	4	22.2	0	0	
Deep soft tissue or internal organs	12	66.7	2	20	
Breast or chest wall	2	11.1	8	80	
At initial diagnosis					
Localized disease	12	66.7	8	80	0.6692
Metastatic disease	6	33.3	2	20	
Primary therapy					
Surgical resection					>0.95
Yes	16	88.9	9	90	
No	2	11.1	1	10	
R-status					0.2421
R0	8	50	8	88.9	
R1	3	18.8	0	0	
R2	1	6.2	0	0	
Unknown	4	25	1	11.1	
Radiotherapy					0.1282
Yes	5	27.8	0	0	
No	13	72.2	10	100	
Chemotherapy and targeted therapy					0.3642
Yes	6	33.3	1	10	
No	12	66.7	9	90	
Chemoradiotherapy					0.3571
Yes	0	0	1	10	
No	18	100	9	90	
Recurrence					0.2543
Yes	8	44.4	7	70	
No	10	55.6	3	30	
Local recurrence	5	27.8	5	50	0.4119
Recurrence therapy					
Surgical resection					>0.95
Yes	6	75	5	71.4	
No	2	25	2	28.6	
Radiotherapy					>0.95
Yes	1	12.5	0	0	
No	7	87.5	7	100	
Chemotherapy and targeted therapy					0.3147
Yes	3	37.5	5	71.4	
No	5	62.5	2	82.6	

Patients with cutaneous AS presented the following sites of origin: the scalp, the temple, the upper leg and presacral. AS of the bone caused painful restriction of mobility and were located in the thoracic vertebras 1–3, caput tibiae, os ilium and suprapatellar. All cardiac AS were located in the right atrium. AS of the mesentery of the small intestine (n = 1) and the adrenal gland (n = 1) were accompanied by spontaneous pain. AS of the thyroid gland appeared as a rapidly growing knotty swelling at the neck. One patient with AS of the deep soft tissue presented with a painful mass in the lower leg. AS of the breast (n = 2) arose from the parenchyma and presented with breast enlargement. In a single case, a bilateral localization was found. In contrast, SAS of the breast were located in the skin in each case and presented as blue-livid discoloration, erythematous plaques, bruise-like macules, blisters, nodules, indurations or exulcerations. The patient with AS of the liver reported about loss of weight, whereas the patient with AS of the thyroid gland presented with a painful mass causing swelling of the head, dyspnea and difficulties in swallowing. The most common metastatic sites at initial diagnosis were the lung (n = 2) and the bones (n = 2) in patients with PAS and the lung (n = 1) together with the skin (n = 1) in patients with SAS.

### Primary therapy, recurrences and their treatment

Table 1 describes the initial treatment, the patterns of failure and their therapy. Surgical resection was the most common primary therapy for both patients with PAS (n = 11; 91.7%) and SAS (n = 7; 87.5%) presenting with localized disease (p > 0.95). Negative surgical margins were achieved in 72.7% of the patients with PAS (n = 8), while all patients with SAS (n = 7; 100%) were curatively resected (p = 0.2451). Among the ones with R0 resection, half of PAS patients (n = 4) received radiotherapy in adjuvant setting, but none of the SAS patients (p = 0.0769). Local recurrence was observed in 33.3% of PAS patients (n = 4) and 50% of SAS patients (n = 4) with nonmetastatic disease at presentation (p = 0.6479). Overall, 61.1% of patients with PAS (n = 11) developed metastases, but only 40% of patients with SAS (n = 4) (p = 0.4328). In terms of metastatic sites, PAS metastasized most frequently into the lung (n = 4), followed by the bones (n = 3) and the lymph nodes (n = 3). In the two latter sites, SAS did not develop metastases. In fact, the skin (n = 2) constituted the most common origin of metastases.

### Chemotherapy and targeted therapy

The most commonly used chemotherapy regimen or targeted therapy agent was the combination of doxorubicin and ifosfamide for patients with PAS (n = 4) and single-agent sorafenib for patients with SAS (n = 5). Four patients with PAS underwent chemotherapy treatment with a combination of doxorubicin and ifosfamide: two had partial remission (PR) and two had progressive disease (PD). Only one patient with SAS receiving these two drugs had PR. Patients with PAS (n = 3) treated with sorafenib had PD, patients with SAS had stable disease (SD) (n = 3) and PD (n = 2). Two PAS patients had SD under the combination of doxorubicin and sorafenib, whereas SAS patients showed SD (n = 1) and PD (n = 1). Both patients with PAS (n = 1) and SAS (n = 1) had complete remission (CR) under treatment with pegylated-liposomal doxorubicin. In the paclitaxel group, one patient with PAS experienced SD, while one patient with SAS showed PD.

### Survival

**Figure 1:**
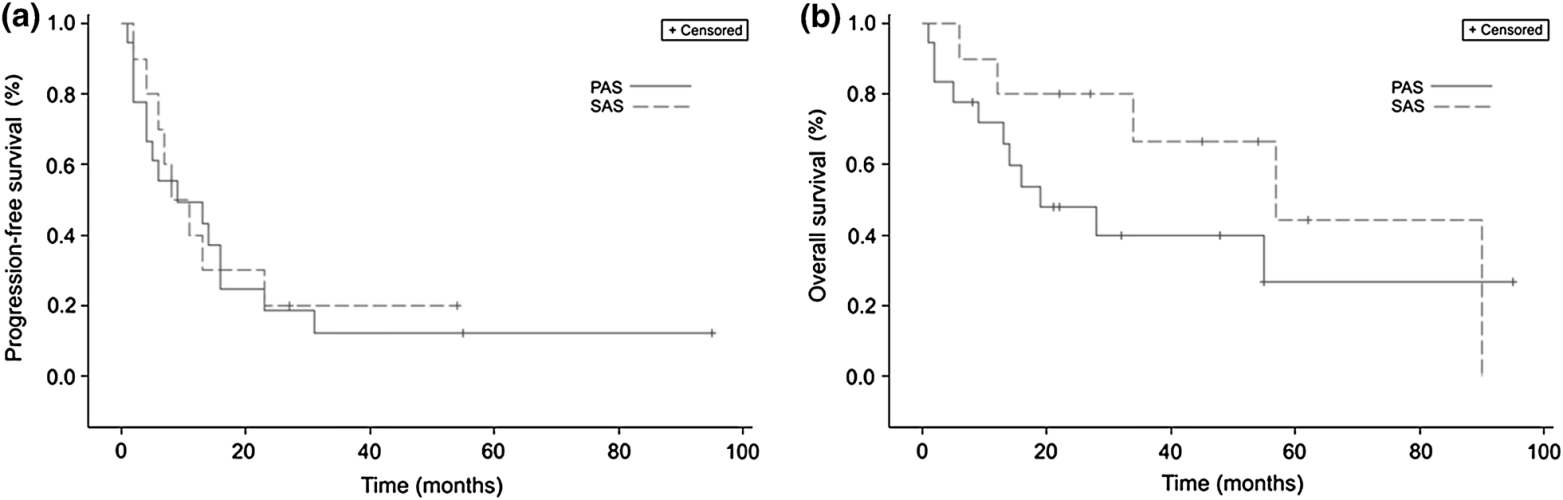
Survival of patients with PAS and SAS. Kaplan–Meier curves for **a** progression-free survival (p = 0.8139) and **b** overall survival (p = 0.2306) for patients with PAS (*solid line*) and SAS (*dashed line*).

The median follow-up was 17.5 months (range 1–95 months) for PAS patients and 39.5 months (range 5–90 months) for SAS patients (p = 0.1235). Eleven patients with PAS (61.1%) have died during the study as opposed to five patients with SAS (50%). Patients with PAS had a median PFS of 9 months (range 1–31 months), whereas patients with SAS relapsed after a median duration of 9.5 months (range 2–23 months) (p = 0.8139) (Figure 1a). Median OS between patients with PAS (19 months; range 1–55 months) and SAS (57 months; range 6–90 months) did not reach statistical significance (p = 0.2306) (Figure 1b). PAS and SAS 5-year OS rate was 6 and 20%, respectively.

Median PFS of PAS and SAS patients who presented with localized disease and were initially treated by surgery was 4 months (range 1–31 months) and 13 months (range 2–23 months), respectively (p = 0.5943). If negative surgical margins were achieved, median PFS was 10 months (range 2–31 months) and 13 months (range 2–23 months) for PAS and SAS patients, respectively (p = 0.6188). PAS and SAS patients presenting with nonmetastatic disease had a median survival from initial diagnosis to appearance of local recurrence of 19.5 months (range 2–31 months) and 10 months (range 2–23 months), respectively (p = 0.3045), whereas median PFS was 4.5 months (range 1–31 months) and 12 months (range 2–23 months), respectively (p = 0.5570).

## Discussion

Primary and secondary AS are rare and aggressive neoplasms. The current analysis of 28 patients compared characteristics of these two subtypes demonstrating both similarities and differences. In this series, overrepresentation of SAS, female preponderance and the high percentage of breast SAS could be seen as a consequence of an increasing use of breast conserving therapy with postoperative radiotherapy in patients with breast carcinoma. That therapeutic procedure is associated with an about 3,200 times increased relative risk to develop SAS [21]. The median latency period from irradiation to diagnosis of SAS was 7.7 years. This fact was similar to findings in previous studies [7, 19]. Besides, the average latency tends to be shorter for breast SAS than for non-breast SAS (6.7 vs. 20.9 years; p = 0.148) [7]. In one case, breast SAS occurred after a latent time interval of 33.5 years being unusual for radiation-induced AS. Such a long period of time is rather reported for other radiation-induced sarcomas [22]. Postirradiation breast sarcomas (excluding AS) are generally diagnosed after a mean latency period of 14.3 years. In particular, radiation-induced breast malignant fibrous histiocytomas and fibroblastic sarcomas develop after a longer latency as opposed to postirradiation breast AS (p < 0.05) [23].

In this series, the percentage of surgery as initial therapy was higher than in previous reports (about 90 vs. 68–75%) [11, 19]. Negative microscopic margins were obtained in 88.9% of SAS patients, but only in half of PAS patients (p = 0.2421). This might be related to surgical methods depending on primary sites. In case of breast AS, R0 resection could be easily achieved through mastectomy (74% [24]). In opposition to that, resection of heart or cutaneous AS causes difficulties (0 and 21.3%, respectively [25, 26]) due to a potential tumor spread around vital structures. Furthermore, median PFS was increased by surgical resection in PAS patients presented with localized tumors when negative microscopic margins were achieved (4 to 10 months). SAS patients were not evaluated because all patients treated by surgery had R0 resection. Jallali et al. [27] found out that curatively resected patients with radiation-induced AS after breast conserving therapy had improved median survival compared to patients with incomplete excision (42 vs. 6 months). In general, surgical margin status has a major impact on patients’ outcome since wide surgical resection with microscopically negative margins is accompanied by significantly prolonged OS and disease-specific survival (DSS), respectively [12, 20].

Radiotherapy constitutes another treatment option, preferably in the adjuvant setting. Mark et al. [13] observed statistically significant difference in 5-year OS between patients treated with and without additional radiotherapy, respectively (54 vs. 19%; p = 0.03). In our series, 27.8% of PAS patients received radiotherapy, but none of SAS patients (p = 0.1282). The latter have already received the maximum dose of radiotherapy in terms of predisposing disease. Nevertheless, in the Riad series, the risk of developing local recurrence was lower in patients with radiation-induced sarcomas, if they were reirradiated after surgical treatment (p = 0.043) [28]. Overall, Buehler et al. [19] analyzed AS patients with localized disease undergoing surgical resection and irradiation. They observed a decreased local recurrence rate (31 vs. 41%) and prolonged time to local recurrence (median 10 vs. 4 months).

In general, patients with inoperable, advanced or metastatic disease are treated by cytotoxic chemotherapy. However, the use is often hampered by the advanced age of patients, their co-morbidities and toxicities. The best outcome in our series was observed with pegylated-liposomal doxorubicin. Skubitz and colleagues [29] reported on six patients receiving this drug but none of them had CR. Three patients had PR, two had SD and one had PD. A study by Italiano evaluated efficacy of doxorubicin and weekly paclitaxel in 117 metastatic AS patients. They found out that response rate of both drugs was higher in patients with SAS as opposed to patients with PAS [16].

An alternative treatment option to conventional chemotherapy represents molecular targeted therapy in the form of angiogenesis inhibition, e.g. by sorafenib or bevacizumab. Maki et al. [18] assessed the efficacy of sorafenib in 145 patients with metastatic or recurrent sarcomas. These included 25 PAS patients (2 PR; response rate 8%) and 9 SAS patients (1 CR, 2 PR; response rate 33.3%). Sorafenib seems to be more active in patients with SAS as opposed to patients with PAS. In a phase II trial from the French Sarcoma Group (GSF/GETO) with 41 advanced AS patients, sorafenib showed a response rate of 23% in chemotherapeutical pretreated population whereas chemotherapy-naive patients had no response [30]. In contrast, no response could be observed in our series, irrespective if pretreated or not.

Our analysis is limited for various reasons. Firstly, the retrospective nature reduced the quality of data since there is no predefined therapeutic algorithm. In fact, AS represent rare and especially heterogeneous neoplasms why this single-center study is primarily descriptive. Furthermore, our small total number of patients was divided into more homogeneous subgroups to counteract limitations of outcome analysis. However, the impact of different treatment options, in particular the role of chemotherapy, targeted therapy and their combination, cannot be addressed adequately in this series and therefore has to be taken into account when interpreting the results. Accordingly, further evaluations with larger study populations are urgently needed.

Median survival from diagnosis to local recurrence tended to be longer in PAS patients compared to SAS patients with initially localized disease (19.5 vs. 10 months; p = 0.3045). According to Abraham et al. [10] the difference is clearly significant, resulting in a higher risk of local recurrence of SAS patients as opposed to PAS patients (p = 0.0001). While median PFS of all PAS and SAS patients showed similar results (9 vs. 9.5 months; p = 0.8139), it tends to be shorter in PAS patients presenting with nonmetastatic disease than in SAS patients (4.5 vs. 12 months; p = 0.5570). This observation might be caused by the more frequent occurrence of metastases in PAS patients during the course of the disease. OS analyses in different subgroups is hampered by both lack of endpoints and the small number of patients. However, AS arising within irradiated tissue in patients with nonmetastatic disease tended to have a shorter OS compared to patients with AS in other sites including lymphedematous fields in the Lindet series (26.5 vs. 45.9 months; p = 0.255). It must be taken into consideration that 22.4 and 62.6% of the entire cohort consist of patients with AS in pre-existing lymphedema and previously irradiated fields, respectively [31]. In particular, the outcome of PAS patients is significantly associated with longer DSS in comparison to SAS patients (p = 0.007) [10]. Different OS was observed between all patients with PAS and SAS in our series (19 vs. 57 months; p = 0.2306). Possible causes of the outcome are the diverse tumor biology, the primary site and the related therapy options. Hung et al. [7] also found no significant difference between PAS and SAS patients. Instead, shorter OS were observed in patients with breast SAS as opposed to patients with non-breast SAS. Moreover, Vorburger et al. [32] observed no statistically difference in terms of OS between patients with PAS and SAS of the breast. Overall, the prognosis remains poor for both PAS and SAS patients, but there seems to be a better perspective for the latter (5-year OS rate 6 and 20%, respectively). Furthermore, median OS of advanced soft-tissue sarcomas was 11.7 months in the Blay series [33]. In view of this fact, SAS belong to the circle of sarcoma entities with the best outcome.

## Conclusion

In conclusion, we have demonstrated that primary and secondary AS constitute not only a rare and thoroughly aggressive, but also a heterogeneous disease. PAS occur at different localizations in the body while the majority of SAS arise from the breast of female patients. SAS show a high local recurrence rate, while PAS tend towards developing metastases. Radical surgical resection remains the mainstay of both primary and recurrence treatment. In future, multicenter prospective randomized trials should investigate new therapeutic strategies like the combination of molecular targeted therapy and cytotoxic chemotherapy. The prognosis is poor for both AS subtypes, but there seems to be a better outcome for SAS. While PFS shows similar results among the two groups, it tends to be shorter in PAS patients presenting with localized disease at initial diagnosis compared to SAS patients. However, subdivision of patients into more homogeneous subgroups is limited by our small number of patients. Also the study’s retrospective nature and the rarity of the disease have an impact on the results which has to be considered. Hence, the relationship of primary and secondary AS has to be investigated, particularly in terms of molecular biology and clinicopathological features, in order to improve the specific treatment options and subsequently the survival.
